# Reduced expression of *brain cannabinoid receptor 1* (*Cnr1*) is coupled with an increased complementary micro-RNA (*miR-26b*) in a mouse model of fetal alcohol spectrum disorders

**DOI:** 10.1186/1868-7083-5-14

**Published:** 2013-08-02

**Authors:** Randa L Stringer, Benjamin I Laufer, Morgan L Kleiber, Shiva M Singh

**Affiliations:** 1 Department of Biology, Molecular Genetics Unit, Western University, London, ON N6A 5B7, Canada

**Keywords:** Cannabinoid receptor 1, Epigenetics, Gene regulation, microRNA, Mouse, Neurodevelopment, Prenatal alcohol exposure

## Abstract

**Background:**

Prenatal alcohol exposure is known to result in fetal alcohol spectrum disorders, a continuum of physiological, behavioural, and cognitive phenotypes that include increased risk for anxiety and learning-associated disorders. Prenatal alcohol exposure results in life-long disorders that may manifest in part through the induction of long-term gene expression changes, potentially maintained through epigenetic mechanisms.

**Findings:**

Here we report a decrease in the expression of *Canabinoid receptor 1* (*Cnr1*) and an increase in the expression of the regulatory microRNA *miR-26b* in the brains of adult mice exposed to ethanol during neurodevelopment. Furthermore, we show that *miR-26b* has significant complementarity to the 3’-UTR of the *Cnr1* transcript, giving it the potential to bind and reduce the level of *Cnr1* expression.

**Conclusions:**

These findings elucidate a mechanism through which some genes show long-term altered expression following prenatal alcohol exposure, leading to persistent alterations to cognitive function and behavioural phenotypes observed in fetal alcohol spectrum disorders.

## Findings

Fetal alcohol spectrum disorders (FASD) describe the continuum of phenotypic effects that may result from prenatal alcohol exposure (PAE). PAE is the most common cause of preventable neurodevelopmental disorders in North America [1,2] and is associated with attention deficit, impaired learning and memory, and hyperactivity [3], as well as an increased risk for anxiety and mood disorders [4]. These cognitive and behavioural changes persist throughout the life of an individual following PAE, though the mechanisms involved in maintaining these life-long changes are not well understood. However, it has been suggested that the effects of PAE may involve long-term changes in gene expression [5] that may be maintained through alcohol-induced epigenetic changes. In particular, we have previously reported that the expression of microRNAs (miRNAs) may be globally altered in the adult mouse brain following PAE [6], which supports recent data by other groups [7,8]. More specifically, these changes in miRNA expression may subsequently alter the expression of target genes, with one miRNA having the potential to regulate many different genes [9]. One such gene may be *cannabinoid receptor 1* (*Cnr1*).

We have previously shown that early neonatal ethanol exposure in mice results in reduced *Cnr1* gene expression in the adult brain [5]. *Cnr1* acts within the endocannabinoid (eCB) system, involved in modulating neurophysiological processes controlling mood, memory, pain sensation, and appetite [10]. *Cnr1* is also thought to be involved in the neuropharmacological effects of alcohol [11] through inhibition of glutaminergic and GABAergic interneurons [12]. Variations in this gene or alterations in its expression are also associated with mood disorders, particularly fear and anxiety phenotypes [13].

Here, we use a C57BL/6J mouse model of binge-like exposure during the period of synaptogenesis [5] to assess a potential relationship between *Cnr1* and its putative regulatory miRNA, *miR-26b*. We evaluated the inverse expression patterns of these two transcripts, hypothesizing that the up-regulation of the miRNA following PAE may in part be responsible for the observed reduction in transcript of a target gene in the adult brain. In these experiments, mice were exposed to two acute doses of alcohol (5 g/kg) at neurodevelopmental times representing the human third trimester equivalent. This method has been previously reported and induces a peak blood alcohol level of over 0.3 g/dL for 4 to 5 hours following injection, and is sufficient to induce neuronal apoptosis and result in FASD-related behaviour [5,14,15]. Our results suggest that ethanol exposure during neurodevelopment may exert its long-term effects by altering the expression of regulatory miRNAs, which may then reduce the expression of a number of target genes that may contribute to the spectrum of phenotypes observed in FASD.

Gene expression data previously was generated through microarray analysis (GEO # GSE34539) of RNA isolated from whole brain tissue of 60-day-old male mice exposed to binge-like levels of alcohol during the third trimester equivalent on postnatal days 4 and 7 (see [5] for methods). miRNA expression array data (GEO # GSE34413) was also generated from the same sample (see [6] for methods). Analysis of these data show a reduction of *Cnr1* (fold change = −1.33, *P* = 6.07 x 10^-5^) in ethanol-treated brains as compared to the saline controls. Also, the miRNA *miR-26b* increased in ethanol-treated mice (fold change = 1.284, *P* = 0.0364) compared to controls.

The potential interaction of the genes and miRNAs identified as differentially expressed by the array studies were analysed using Ingenuity’s® Micro-RNA Target Filter. This analysis identified *miR-26b* as a high-confidence predicted regulator of *Cnr1* expression.

**Figure 1:**
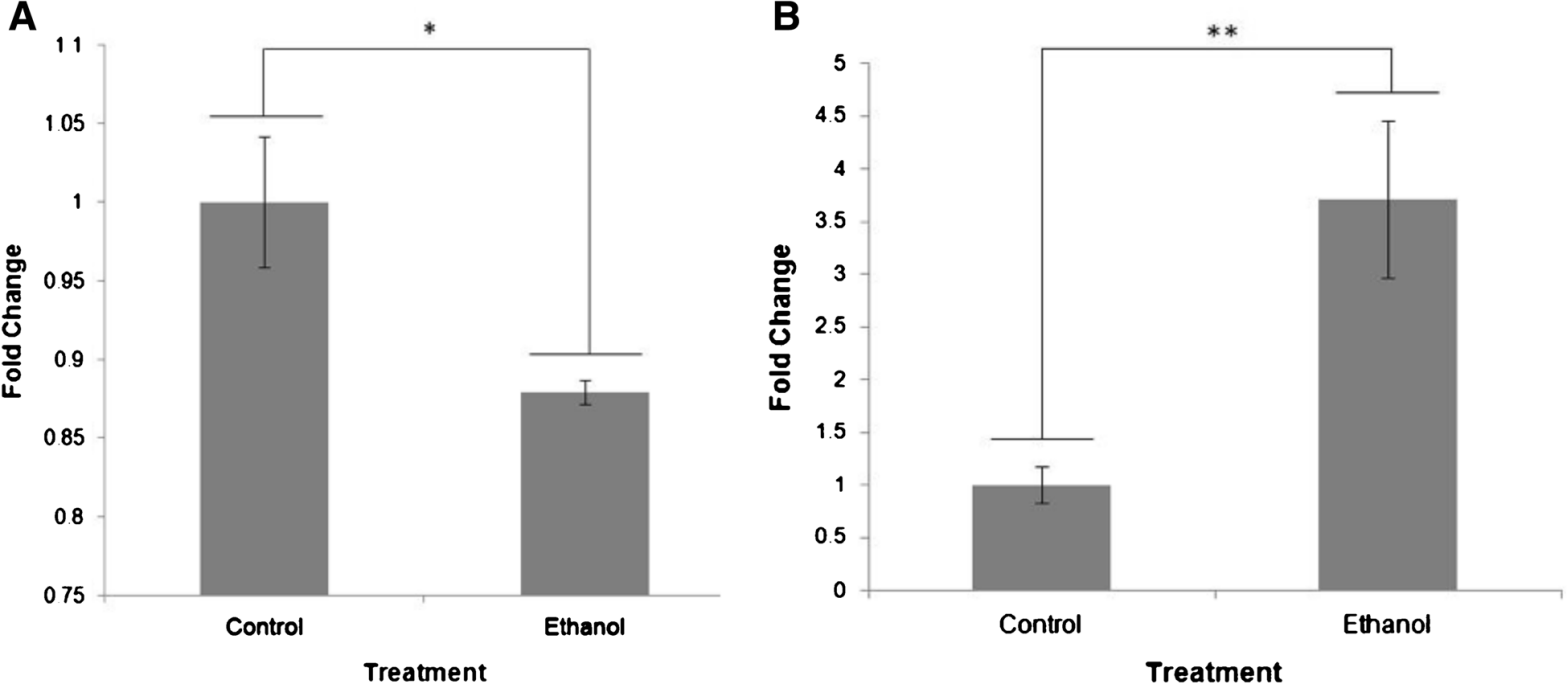
**Analysis of Gene and miRNA expression via qPCR. (A)** Change in *Cnr1* mRNA levels in male control and alcohol-treated whole brain samples normalized to control. This figure was reproduced with permission from the authors [5]. **(B)** Change in *miR-26b* levels in male control and alcohol-treated whole brain samples normalized to control. Data are fold change ± SEM. Control n = 5, alcohol n = 5. *P <0.01, **P <0.05.

**Figure 2:**
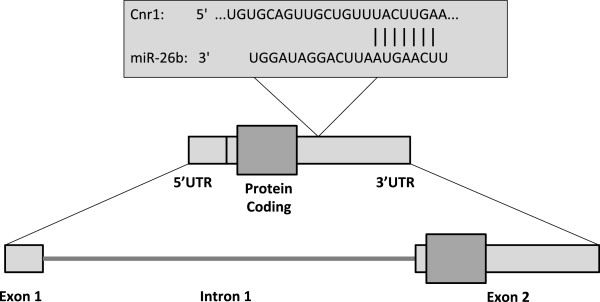
**TargetScan® analysis of *miR-26b*-*Cnr1 *binding sites provides a model for *Cnr1 *and *miR-26b *interaction.** The seed region of *miR-26b* has the potential to bind to the 3’-UTR of the *Cnr1* transcript.

The reduction of *Cnr1* transcript was confirmed by real time RT-PCR [5], showing a 1.14-fold decrease in expression in ethanol-treated male brains as compared to matched controls (*P* = 0.004; Figure 1A). Further, we demonstrated a significant increase in the level of *miR-26b* miRNA in ethanol-treated samples (fold change = 3.71, *P* = 0.012) compared to matched controls (see [6] for methods) (Figure 1B). This inverse relationship within the same sample set suggests that the two observations may be biologically related. This potential interaction was further analysed using the TargetScan® Human 6.2 predictor for miRNA targets [16], which shows that the seed region of *miR-26b* possesses complementarity to the 3’-UTR of the *Cnr1* transcript and has a significant potential to bind this region (Figure 2). The probability of conserved targeting (P_CT_) analyses the preferential conservation of binding sites [16]. It has the advantage of identifying targeting interactions that are not only more likely to be effective but also those that are more likely to be consequential for the animal, given the evolutionary conservation. The analysis calculated a P_CT_ score of 0.84, which indicates a significant degree of confidence in the predicted interaction. Next, we evaluated expression of *Cnr1* and *miR-26b* to confirm their relative expression levels.

*miR-26b* is encoded from an intron of small C-terminal domain phosphatase [17]. Interestingly, it is involved in neuronal differentiation as its transcription results in a negative feedback loop that is absent in neural stem cells [18]. *miR-26b* has also been shown to regulate the expression of brain-derived neurotrophic factor (*BDNF*), a gene strongly implicated in neurodevelopment and related disorders (i.e., schizophrenia) [19], including the effects of PAE [5].

This altered expression of *miR-26b* may have the ability to affect downstream gene expression by binding to the mRNA transcripts of its target genes. We have demonstrated that *miR-26b* shows complementarity to a region of the 3’-UTR of the *Cnr1* transcript (Figure 2), which gives it the potential to regulate the expression of *Cnr1*. This regulation by miRNAs generally occurs through blocking of translation and/or promoting degradation of the target transcript [9]. The up-regulation of *miR-26b* correlates with the reduced *Cnr1* transcript observed in the adult brain of mice neurodevelopmentally exposed to alcohol. [7] Our results suggest that this regulatory mechanism also occurs *in vivo*, and that the stable alteration of miRNA as a result of neurodevelopmental teratogenesis may affect long-term gene expression of its target transcript(s) long after exposure.

It is possible that relationships such as these may have the ability to influence the aberrant behavioural phenotypes seen in FASD. The eCB system, for instance, plays a strong role in anxiety-related behaviour [20], which has been shown to increase in adult mice following PAE [21]. Previous studies evaluating *Cnr1* knockout mice have demonstrated increased anxiety-like phenotypes [13]. This suggests that the observed reduction in *Cnr1* expression demonstrated here may contribute to our observation of anxiety-like behaviour following PAE.

Ultimately, these findings provide a mechanism by which the long-term change in *Cnr1* expression is maintained following PAE. They also suggest that the alteration of neurodevelopmentally-important miRNAs can influence the long-term function of biological pathways that influence cognition and behaviour. Epigenetic regulators of gene expression may then be affected by PAE, subsequently exerting pleiotropic effects on numerous gene targets that then contribute to the long-term and variable neurobehavioural effects associated with FASD.

## Abbreviations

Cnr1: Cannabinoid receptor 1; eCB: Endocannabinoid; FASD: Fetal alcohol spectrum disorders; miRNA: microRNA; PAE: Prenatal alcohol exposure.

## Competing interests

The authors declare no competing financial interests.

## Authors’ contributions

This project was developed by RLS, BIL, MLK, and SMS. MLK raised the mice, performed the experimental interventions, and extracted the RNA. RLS performed the qPCR reactions. BIL and RLS performed the bioinformatic analysis. RLS, BIL, MLK, and SMS wrote the manuscript. All authors read and approved the final manuscript.
